# Knowledge, Attitudes, and Availability of Romanian Firefighter Paramedics to Adopt the Role of Community First Responders in Out-of-Hospital Cardiac Arrest

**DOI:** 10.3390/jcm15145526

**Published:** 2026-07-15

**Authors:** Paul Lucian Nedelea, Alexandra Haută, Vlad Constantin Donica, Mihaela Corlade-Andrei, Radu-Alexandru Iacobescu, Tudor Ovidiu Popa, Carmen Diana Cimpoeșu

**Affiliations:** 1 Grigore T. Popa University of Medicine and Pharmacy Iasi, 700115 Iasi, Romania; paul-lucian.nedelea@umfiasi.ro (P.L.N.); vlad-constantin.donica@umfiasi.ro (V.C.D.); mihaela.corlade2@umfiasi.ro (M.C.-A.); radu_iacobescu@yahoo.com (R.-A.I.); tudor.popa@umfiasi.ro (T.O.P.); carmen.cimpoesu@umfiasi.ro (C.D.C.); 2 Emergency Care Department, Sf. Spiridon University Emergency Hospital, 700111 Iasi, Romania; 3 Thoracic Surgery Department, Respiratory Disease Hospital, 700115 Iasi, Romania

**Keywords:** out-of-hospital cardiac arrest, first responder, emergency medical services, pre-hospital emergency care

## Abstract

**Background:** Out-of-hospital cardiac arrest (OHCA) remains a major public health challenge in Romania. Although trained first-responder systems have improved prehospital response in other European settings, Romania lacks a structured national framework. This study assessed the knowledge, attitudes, perceived preparedness, and willingness of Inspectorate for Emergency Situations (ISU) personnel to participate as community first responders outside working hours. **Methods:** A national cross-sectional survey was conducted using a structured online questionnaire distributed to operational ISU personnel. The final sample included 1537 participants from five professional categories: firefighter officers with paramedic training, firefighter sub-officers with paramedic training, firefighter officers, firefighter sub-officers, and other personnel. Multivariable binary logistic regression was used to identify factors associated with high enthusiasm and desire for additional training. **Results:** More than 70% of respondents supported the integration of firefighter-paramedics into a community first-responder system, and over 75% expressed willingness to participate. Significant differences were observed between professional categories in demographic, professional, and first responder-related variables. In multivariable analysis, firefighter officers with and without paramedic training, as well as other personnel, had higher odds of high enthusiasm compared with firefighter sub-officers without paramedic training. Desire for additional training was independently associated with female sex and firefighter officer status, with or without paramedic training. **Conclusions:** Romanian ISU personnel showed favorable self-reported attitudes and high willingness to participate in a structured OHCA first-responder system. However, these findings reflect perceived motivation rather than operational feasibility, competence, or improved patient outcomes. Implementation would require standardized training, legal clarification, dispatch integration, adequate AED access, supervision, audit systems, and prospective evaluation using patient-level OHCA outcomes.

## 1. Introduction

Out-of-hospital cardiac arrest (OHCA) remains one of the most critical challenges for public health systems worldwide, characterized by a high incidence and a mortality rate that demands immediate and coordinated intervention measures. At the European level, statistical data reveal a considerable variability in incidence, estimated between 67 and 170 cases per 100,000 inhabitants [1,2]. Correlatively, patient survival to discharge reflects the efficiency or deficiencies of the emergency medical infrastructure, oscillating between discouraging values of below 5% in certain regions and over 20% in communities with optimized response systems [1]. In Romania, the current situation indicates a survival rate that consistently remains below the 10% threshold, a worrying indicator that suggests the existence of major dysfunctions within what specialists call the “chain of survival,” particularly in the early stages of the critical event [3,4].

The concept “chain of survival” was first introduced by Mary Newman in 1989 and was subsequently further developed and refined by Cummins and colleagues in 1991 through the periodic guidelines of the European Resuscitation Council, representing the theoretical pillar of emergency assistance, emphasizing the synchronization of four essential links [5]. This sequence begins with the early recognition of cardiac arrest signs and alerting emergency services, continues with the immediate initiation of cardiopulmonary resuscitation (CPR) and rapid defibrillation, culminating in high-specialty post-resuscitation care [3,6]. The critical importance of the first two stages cannot be underestimated, given that the time factor represents the main determinant of the vital prognosis. Clinical studies confirm that every minute of delay in applying resuscitation maneuvers reduces the probability of survival by a percentage between 7% and 10% [3] and a decision to transport OHCA patients in emergency care should be made in the first 15 min after arrival [7]. Furthermore, if automated external defibrillation (AED) is performed within the first three to five minutes of collapse, survival rates for shockable rhythms can reach impressive values between 50% and 70% [8]. Unfortunately, in the Romanian medical landscape, bystander intervention is often absent or occurs too late to change the victim’s clinical course.

In response to the need to shorten intervention times, the concept of the “first responder” has developed internationally over the last two decades. This strategy is based on the use of modern technology, specifically mobile geolocation applications, to mobilize individuals trained in basic life support who happen to be in the proximity of a victim [9,10,11]. These volunteers have the capacity to initiate resuscitation maneuvers and use an AED in the critical interval between the call to the emergency number and the arrival of the ambulance [12]. Experiences documented in states such as the Netherlands or Denmark demonstrate that integrating trained citizens into digital alerting networks has led to an increase in the early intervention rate to over 60%, a fact that has significantly improved long-term survival indicators [13,14].

Despite these documented successes, the implementation of first responder systems remains marked by pronounced heterogeneity at the European level. In Romania, this endeavor is still in an incipient phase, limited to pilot projects or fragmented local initiatives, without benefiting from a coherent national framework. Major obstacles to the large-scale adoption of this model include legislative uncertainty regarding the legal liability of individuals providing first aid, the absence of an integrated digital infrastructure within emergency dispatch centers, and a low density of automated external defibrillators in public spaces [15,16,17]. These barriers, correlated with an unequal distribution of medical resources between urban and rural environments, prolong response times and drastically limit the effectiveness of intervention in the first minutes, which are often decisive.

In this context of necessary reform, paramedical personnel represent a strategic resource of invaluable worth. These professionals possess not only solid theoretical knowledge in life support but also vast practical experience in managing crisis situations, being already integrated into the operational structures of emergency services. Due to their training, paramedics can provide an effective bridge between the moment the arrest occurs and the arrival of the advanced medical team. However, their potential to act on a voluntary basis outside of official working hours through early warning systems remains largely unexplored in Romania. To date, specialized literature has focused mainly on institutionalized intervention, neglecting the study of psychological, organizational, or legal barriers that might influence the desire of paramedics to get involved in such a civilian intervention system.

In Romania, a firefighter paramedic is a firefighter employed within ISU, which operates in close institutional collaboration with SMURD (Mobile Emergency Service for Resuscitation and Extrication). Their scope of practice includes: clinical assessment and patient monitoring, CPR with AED, bag-valve-mask ventilation, and wound/trauma management. Medication administration requires physician authorization. Peripheral IV access is available to those who have completed the advanced training module, but it is not universal. Paramedic-certified ISU firefighters rotate on SMURD Basic Life Support crews, alternating these duties with standard firefighting shifts within the same institution. As first responders in the proposed community model, they would act voluntarily and outside working hours, responding via a mobile alert application when in proximity to a cardiac arrest victim, and providing basic life support until a formal medical crew arrives.

The present research aims to address exactly this information gap by systematically evaluating the level of knowledge, attitudes, and actual availability of Romanian paramedics to adopt the role of first responders. The fundamental hypothesis from which this endeavor starts is that, although there is a solid base of professional skills and a general favorable attitude toward helping victims, practical implementation is inhibited by structural and legislative factors. The research objectives aim for an in-depth analysis of how these professionals perceive the risks and benefits of participating in mobile alerting networks, identifying the main obstacles that must be eliminated to facilitate this process.

By achieving these objectives, the study will not only provide a clear picture of the readiness of paramedical personnel but will also contribute to the substantiation of public policy recommendations. Optimizing the management of OHCA by activating a national network of qualified first responders represents, ultimately, the only viable strategy for improving survival chances and aligning Romania’s emergency system with European performance standards.

## 2. Materials and Methods

This section details the methodological framework used to conduct the study regarding the role of the Inspectorate for Emergency Situations (ISU) personnel within a first-responder-type primary care system in Romania. The research was specifically designed to evaluate the availability, knowledge, and attitudes of operational staff toward the possibility of voluntary involvement in providing first aid to patients in cardiorespiratory arrest outside of normal working hours, within the context of developing such systems at a European level where volunteers are alerted via specialized applications. The study was conducted at a national level, targeting firefighting units within the Inspectorate for Emergency Situations throughout Romania. Data were collected via the Google Forms online platform, a method that allowed easy access for participants from various counties and ensured secure data management in accordance with European data protection requirements.

The primary instrument was a structured questionnaire aimed at collecting essential information regarding demographics and professional experience, expressed in years of activity within the institution and specifically in years of experience as a paramedic. The questionnaire was distributed online to operational ISU personnel across Romania. Participation was voluntary and anonymous. Eligible respondents were active ISU personnel belonging to one of the predefined professional categories: firefighter officers (FO) with paramedic training, firefighter sub-officers (FSO) with paramedic training, FO, FSO, and other personnel. Only complete responses were included in the final analysis. 

Furthermore, professional training was evaluated by indicating the completion of first aid courses. The perception of the first responder role was measured through opinion scales regarding the appropriateness of personnel involvement in this system, the active availability to participate in community interventions, and the perceived level of readiness for managing emergency situations. The questionnaire also investigated the level of enthusiasm on a scale of one to ten, as well as the qualitative identification of the main obstacles or challenges in integrating paramedic firefighters into such a national project. The resulting data were collected and organized into a database using Microsoft Excel, subsequently facilitating the comparative analysis between the three evaluated models. Examples of the questionnaire items are attached as a supplementary file.

The final research sample included a total of 1537 participants recruited from operational personnel performing activities within the ISU across Romania. These were segmented into five distinct professional categories to allow for rigorous comparative analysis, including: 48 FO with paramedic training, 948 FSO with paramedic training, 169 FO, 281 FSO, and 91 participants from other personnel categories.

Statistical analysis was performed using the SPSS program (IBM SPSS Statistics, version 26). The statistical analysis methodology involved evaluating the distribution of all continuous variables through the Kolmogorov–Smirnov and Shapiro–Wilk tests. Due to significant deviations from normality for most groups and variables (such as age, experience, and readiness scores), non-parametric statistical methods were applied exclusively for all subsequent analyses. Differences between personnel categories were evaluated using the Kruskal–Wallis test to assess perception, involvement, and enthusiasm. To identify specific differences between pairs of groups, post hoc comparisons were performed using the Mann–Whitney U test, applying the Bonferroni correction to adjust the significance threshold to a value of *p* = 0.005. Multivariable binary logistic regression was performed to identify independent factors associated with high enthusiasm and desire for additional training. The reference category for the staff category was firefighter sub-officer without paramedic training. High enthusiasm was defined as an enthusiasm score ≥8. Desire for training was defined as a training score ≥4. Age was entered per 10-year increase and ISU experience per 5-year increase. Both models presented statistically significant results and have acceptable calibration by Hosmer–Lemeshow testing; however, their explanatory power is limited, especially for enthusiasm.

The research was conducted in strict compliance with ethical standards regarding the protection of natural persons with regard to the processing of personal data, in accordance with EU Regulation No. 679/2016 (GDPR) as well as with the Declaration of Helsinki, and approved by the Institutional Review Board of Grigore T. Popa University of Medicine and Pharmacy Iasi, Romania, no. 521/25 January 2025. Participation was entirely voluntary, and the completion of the questionnaire represented the subjects’ implicit consent to participate in the study. Confidentiality was guaranteed by the fact that the information obtained does not allow for the individual identification of respondents, as providing a name was not required for participation. Statistical processing was carried out only at the group level, and results are not presented individually in any scientific publication, thus ensuring a high standard of professional integrity and respect for the participants. All collected data were stored securely on the online platform used, providing a solid methodological framework for evaluating the feasibility of implementing the first aid project in Romania.

## 3. Results

The current research has generated a substantial volume of data providing a clear perspective on the demographic dynamics and professional attitudes within the Inspectorate for Emergency Situations (ISU), in the context of the proposed implementation of a “first responder” qualified first-aid system. The statistical evaluation started from a sample of 1537 participants distributed across different categories, which allowed for a deep comparative analysis.

The sample configuration is numerically dominated by FSO with paramedic training, representing over 60% of the total participants, followed by FSO without this specialization and FO. A primary relevant observation concerns the gender distribution, where a massive male predominance of over 95% is noted. Although women represent a minority of approximately four percent, their presence is distributed across all staff categories, albeit with an extremely reduced representation among paramedic officers. This structure reflects the traditional profile of intervention forces but also raises interesting questions regarding differences in perception between genders.

The average age of the entire study group is around 36 years; however, the sub-category analysis reveals a clear fragmentation as seen in Table 1. The highest average ages were identified among FSO, while the auxiliary personnel group is considerably younger, with an average of approximately twenty-four years. These differences in age are closely correlated with years of activity within the institution. Officers with paramedic training stand out with the longest professional history, accumulating an average of nearly fifteen years of experience in the ISU. In contrast, specific experience in paramedic activity varies drastically, being almost non-existent in non-paramedic groups and reaching maximum levels of approximately nine years among specialized officers.

Officers with paramedic training reported the highest levels of enthusiasm, involvement, and perceived readiness. In contrast, sub-officers and non-firefighter personnel presented comparatively lower scores, particularly in the areas of theoretical and practical preparedness. The feeling of being “prepared” to manage critical situations is distributed heterogeneously; while the majority feel at least partially prepared, there is a minority segment reporting a total lack of preparation, located primarily in areas without direct exposure to paramedic activity. A remarkable aspect is the massive desire for additional training, with over sixty-five percent of participants requesting more detailed courses to improve their action skills.

The use of the Kruskal–Wallis test demonstrated that membership in a specific staff category exerts a statistically significant influence on all dimensions evaluated. Differences are evident not only in demographic data but also in subjective perceptions. Post hoc comparisons conducted via the Mann–Whitney U test with Bonferroni correction revealed notable disparities between officers and sub-officers, with significant results presented in Table 2. Officers consistently obtained higher scores in indicators of involvement, perceived readiness, and enthusiasm compared to sub-officers, with the effect size being small to moderate.

The direction of significant pairwise differences was established according to the mean ranks. In most comparisons, the first-listed group showed higher mean ranks, reflecting higher item scores. Exceptions were observed for FSO + paramedic vs. FO, where FO scored higher for “firefighters as first responders” and “involvement as first responder,” and for FSO vs. Others, where Others scored higher for “first aid course.”

Multivariable binary logistic regression was performed to identify independent factors associated with high enthusiasm and desire for additional training (Table 3).

The model for high enthusiasm was statistically significant (χ^2^ = 40.028, df = 8, *p* < 0.001), with acceptable calibration according to the Hosmer–Lemeshow test (χ^2^ = 3.218, df = 8, *p* = 0.920), although explanatory power was limited (Nagelkerke R^2^ = 0.035). Compared with FSO without paramedic training, FO with paramedic training had higher odds of high enthusiasm (aOR = 3.46, 95% CI: 1.62–7.39, *p* = 0.001), as did FO without paramedic training (aOR = 2.57, 95% CI: 1.69–3.90, *p* < 0.001) and other personnel (aOR = 2.08, 95% CI: 1.22–3.53, *p* = 0.007). Age, ISU experience, female sex, FSO paramedic status, and completion of first aid training were not independently associated with high enthusiasm.

The model for desire for training was also statistically significant (χ^2^ = 90.285, df = 8, *p* < 0.001), with acceptable calibration (Hosmer–Lemeshow χ^2^ = 8.176, df = 8, *p* = 0.416) and limited explanatory power (Nagelkerke R^2^ = 0.079). Female participants had higher odds of expressing desire for additional training (aOR = 2.21, 95% CI: 1.11–4.42, *p* = 0.024). Compared with FSO without paramedic training, FO with paramedic training (aOR = 4.14, 95% CI: 1.74–9.82, *p* = 0.001) and FO without paramedic training (aOR = 4.08, 95% CI: 2.44–6.82, *p* < 0.001) had higher odds of requesting training. Age showed a borderline association but did not reach statistical significance (aOR = 0.80, 95% CI: 0.63–1.00, *p* = 0.053).

## 4. Discussion

The present study represents one of the first national-scale inquiries into the subjective readiness of Romanian ISU personnel to assume the role of community first responders in OHCA. A previous assessment of the level of preparation of Romanian emergency healthcare workers in disaster response provided moderate levels of readiness with insufficient training and practice. This aspect highlights the need for modern training programs, as well as improving collaboration with other emergency responders [18]. Our findings indicate favorable self-reported attitudes and high willingness among ISU personnel, although these subjective indicators should not be interpreted as proof of institutional or operational readiness.

The broad-based willingness to participate, exceeding 75% of the sample, is consistent with data reported in comparable European studies. In the Netherlands and Denmark, the mobilization of trained volunteers through mobile geolocation platforms contributed to bystander intervention rates surpassing 60%, with measurable improvements in patient survival [13,14]. Romania’s ISU workforce, with its established paramedic training and operational experience in emergency situations, may represent an important candidate group for such a system because of its existing emergency response experience and institutional structure. However, direct comparison with lay volunteer systems was beyond the scope of this study.

While most studies identified a relationship between the intervention of first responders and higher rates of recovery in OHCA patients [19], others found no significant differences in improved survival rates [20]. Regardless, a faster response time was observed. Such data has given rise to programs in which emergency medical services are dispatched at the same time as first responders, increasing survival to 30 days compared to only trained medical personnel [21].

The present findings should be interpreted in the context of existing Romanian patient-level OHCA evidence. Ichim et al. analyzed 508 OHCA cases and reported high mortality, lower ROSC rates in rural compared with urban patients, and better resuscitation outcomes in patients with shockable rhythms and lower cumulative adrenaline doses [22]. These data highlight the clinical and system-level challenges of OHCA management in Romania. While their study evaluated patient outcomes, the present study evaluates personnel attitudes and perceived preparedness. Prospective studies are needed to determine whether such integration improves operational indicators and patient-level outcomes.

A central study finding is the prominent role of self-perceived preparedness as a driver of enthusiasm. Self-perceived preparedness differed significantly between professional categories and remains an important descriptive finding. However, in the adjusted multivariable model, the main independent predictors of high enthusiasm were professional category variables rather than preparedness-related subjective measures. Therefore, preparedness should be interpreted as an important perceived barrier or facilitator, but not as an independently confirmed predictor in the final model. This finding aligns with the broader literature on voluntary emergency response, which consistently identifies self-efficacy as a prerequisite for sustained participation [23,24]. Importantly, the desire for additional training was present across all staff categories, suggesting that perceived gaps in preparedness are recognized and that targeted educational interventions could substantially enhance operational readiness. Female participants had independently higher odds of expressing desire for additional training. Age showed only a borderline association and should therefore be interpreted cautiously.

A pillar of the research was evaluating the personnel’s conviction that firefighter paramedics should be officially integrated into a qualified community first-aid system. The data indicate generalized support, with over 70% of respondents expressing agreement or total agreement with this vision. Interestingly, although differences between categories exist, the desire for active participation in community emergency interventions is very high, with over three-quarters of the sample stating they would “probably” or “certainly” like to be involved. This enthusiasm, however, is not uniform, being significantly influenced by professional status and previous training.

The hierarchical differences observed between officers and sub-officers merit specific attention. Officers with paramedic training consistently outperformed other groups on all attitudinal dimensions, a pattern likely attributable to the combination of advanced training, greater institutional seniority, and increased exposure to high-acuity emergency scenarios. Conversely, sub-officers, despite constituting the operational majority, displayed comparatively lower scores in perceived readiness and enthusiasm. This discrepancy may reflect differences in role expectations, operational exposure, perceived responsibility, or confidence in autonomous decision-making. Further qualitative and competency-based studies would be needed to determine whether current training frameworks adequately support sub-officers for autonomous community intervention.

Paramedic sub-officers constitute the operational core; they sometimes present lower perceived readiness scores than their officers. This may indicate greater pressure felt at the execution level or a more acute awareness of field limitations. When comparing FO with personnel in the “Others” category, the significant difference was maintained only regarding the general perception of the first responder role, while for the desire for training or involvement, the differences were no longer as sharp after applying statistical corrections. The results highlight substantial self-reported willingness among respondents, but operational readiness would require objective assessment of skills, availability, dispatch integration, legal protection, and long-term participation. Although the staff structure is diverse, there is a common denominator represented by the desire to contribute to community safety through qualified first-aid interventions. However, the success of such a program depends on carefully calibrating training, given that needs differ; younger individuals and women request more training, while sub-officers could benefit from programs aimed at increasing their confidence in their own intervention capacities.

The disparities identified between categories suggest that the implementation strategy cannot be universal but must take into account the specific experience of each group. Although paramedic background and first aid training differed substantially between professional categories, the adjusted model showed that professional category, rather than first aid course completion alone, was independently associated with high enthusiasm. These data provide the necessary foundation for administrative policies that can transform employees’ desire for involvement into an efficient and professional community-first-aid operational system.

Beyond individual attitudes, the structural challenges facing the implementation of a first responder system in Romania are substantial. Legislative ambiguity surrounding the legal liability of individuals providing first aid outside their professional hours remains a critical deterrent [15,16,17]. The absence of a dedicated national digital alerting platform integrated with emergency dispatch centers further limits feasibility. Additionally, the uneven geographic distribution of AEDs, particularly in rural and peri-urban areas, undermines the operational value of a volunteer network. These systemic deficiencies must be addressed concurrently with attitudinal change if the transition from individual willingness to functional community response is to be achieved.

International experience suggests that legal liability can be addressed by formally defining the responder’s status during authorized activation. In England, Community First Responders have been required to sign agreements before responding on behalf of ambulance services, and liability protection has been provided when responders act as agents of the ambulance service [25]. France offers another model through the legal status of “citizen rescuer,” which recognizes emergency assistance as a public-service-related action and limits civil liability except in cases of gross or intentional fault [26]. These examples suggest that Romanian off-duty ISU first responder activity should not be treated as informal volunteering, but could be structured as an authorized extension of the public service responder role. Under such a model, ISU personnel would retain their primary duties while also being eligible for after-hours or on-call activation through an official dispatch or digital alerting platform. Liability coverage, supervision, and operational accountability would apply only during authorized activations, supported by documented training, clear protocols, audit mechanisms, and possibly response-based compensation or formal recognition.

### Limitations

Several limitations must be acknowledged when interpreting the findings of this study. First, the cross-sectional design precludes causal inference; the associations identified between attitudinal variables and enthusiasm represent correlations that may be influenced by unmeasured confounding factors. Longitudinal follow-up studies would be necessary to assess whether expressed willingness translates into sustained participation over time.

Second, data collection via a self-administered online questionnaire introduces the possibility of social desirability bias. Participants may have overstated their willingness to volunteer or their perceived level of preparedness, given the professional context in which the survey was administered. This may partially explain the high rates of reported willingness to participate, which should be verified through objective skill assessments and simulated intervention scenarios. Another aspect is that the questionnaire was developed for the purposes of this study and was not formally validated or pilot-tested before national distribution. Reliability metrics, such as internal consistency coefficients, were not calculated. Although the full questionnaire is provided as a Supplementary File S1 for transparency, the absence of formal validation may limit the comparability and reproducibility of the findings.

Third, the study population is exclusively composed of ISU personnel, limiting the generalizability of findings to other categories of potential first responders, such as police officers, military personnel, or community health workers. A broader, multi-sector analysis would provide a more comprehensive picture of Romania’s first responder potential.

Fourth, the predominantly male composition of the sample restricts the statistical power of gender-related analyses, and the significant findings regarding women should be interpreted with caution given the small absolute numbers involved.

In addition, although multivariable regression was performed, the explanatory power of the models was limited, as reflected by low Nagelkerke R^2^ values. Therefore, other unmeasured factors, such as previous OHCA exposure, confidence in AED use, perceived legal risk, workload, geographic location, and willingness to respond outside working hours, may also influence enthusiasm and training demand.

Finally, the absence of OHCA outcome data (return of spontaneous circulation, survival to hospital discharge) within this study design prevents direct correlation between the attitudes measured and actual patient outcomes. Future research integrating registry data with personnel attitude surveys would substantially strengthen the evidence base for policy recommendations.

## 5. Conclusions

This study suggests that Romanian ISU personnel represent a professionally relevant and highly willing group for potential inclusion in a structured national first responder network for OHCA. The findings, based on 1537 respondents across professional categories, indicate favorable self-reported attitudes toward community-based emergency response and support the need for further development of training, legal, and operational frameworks.

However, willingness alone should not be interpreted as proof of operational feasibility, sustained engagement, clinical competence, or improved OHCA outcomes. Translation into practice would require differentiated training programs, clear operational protocols, legal protection for off-duty interventions, integration with emergency dispatch systems, expanded AED availability, supervision, and audit mechanisms. Future prospective studies should evaluate whether such a model improves response time, bystander CPR, AED use, ROSC, survival to discharge, and neurological outcomes.

Therefore, the integration of ISU personnel into a technology-enabled first responder system may represent a promising direction for Romanian emergency care policy, but its effectiveness must be confirmed through structured implementation and patient-level outcome evaluation.

## Figures and Tables

**Table 1:** Descriptive characteristics by staff category: median [interquartile range] or percentage (%).

Variable	FO + Paramedic (*n* = 48)	FSO + Paramedic (*n* = 948)	FO (*n* = 169)	FSO (*n* = 281)	Others (*n* = 91)
Age (years)	38.5 [7]	38.0 [16]	38.0 [11]	40.0 [9]	23.0 [3]
ISU experience (years)	17 [5]	16 [13]	14 [11]	16 [14]	2 [1]
Paramedic experience (years)	10 [12]	7 [9]	0 [0]	0 [4]	0 [0]
Male gender (%)	94%	98%	91%	91%	99%
First aid course (%)	100%	~100%	70%	50%	69%
Firefighters as first responders	4 [1]	4 [1]	4 [1]	4 [1]	4 [2]
Involvement as first responder	5 [1]	4 [1]	5 [1]	4 [3]	4 [1]
Preparedness	4 [1]	4 [1]	4 [1]	3 [2]	4 [1]
Training level	4 [0]	4 [2]	4 [0]	4 [2]	4 [0]
Enthusiasm	9 [2]	8 [4]	8 [3]	7 [4]	8 [2]

FO—firefighter officer; FSO—firefighter sub-officer; FO + paramedic, firefighter officer with paramedic training; FSO + paramedic, firefighter sub-officer with paramedic training.

**Table 2:** Post hoc pairwise comparisons were performed using the Mann–Whitney U test with Bonferroni correction (adjusted significance level *p* < 0.005).

Comparison	Variable	Z	*p*-Value	Effect Size (r)
FO + paramedic vs. FSO + paramedic	Firefighters as first responders	−4.48	<0.001	0.14
	Involvement	−3.30	0.001	0.10
	Training	−3.29	0.001	0.10
	Enthusiasm	−4.40	<0.001	0.14
FO + paramedic vs. FO	First aid course	−4.29	<0.001	0.29
	Preparedness	−3.91	<0.001	0.26
FO + paramedic vs. FSO	All variables	≤−3.18	<0.001	0.20–0.36
FO + paramedic vs. Others	First aid course	−4.29	<0.001	0.36
	Preparedness	−4.25	<0.001	0.36
	Enthusiasm	−3.23	0.001	0.27
FSO + paramedic vs. FO	All variables	≤−3.40	<0.001	0.11–0.49
FSO + paramedic vs. FSO	First aid course	−22.82	<0.001	0.65
	Preparedness	−8.40	<0.001	0.24
FO vs. FSO	All variables	≤−3.04	≤0.002	0.14–0.31
FO vs. Others	Firefighters as first responders	−3.18	0.001	0.20
FSO vs. Others	First aid course	−3.23	0.001	0.17

FO—firefighter officer; FSO—firefighter sub-officer; FO + paramedic, firefighter officer with paramedic training; FSO + paramedic, firefighter sub-officer with paramedic training.

**Table 3:** Multivariable logistic regression analysis of factors associated with high enthusiasm and desire for training.

Variable	High Enthusiasm aOR (95% CI)	*p*-Value	Desire for Training aOR (95% CI)	*p*-Value
Age, per 10-year increase	1.03 (0.83–1.28)	0.793	0.80 (0.63–1.00)	0.053
ISU experience, per 5-year increase	1.03 (0.90–1.18)	0.685	0.95 (0.83–1.09)	0.472
Female gender	1.49 (0.85–2.60)	0.166	2.21 (1.11–4.42)	0.024
FO + paramedic vs. FSO	3.46 (1.62–7.39)	0.001	4.14 (1.74–9.82)	0.001
FSO + paramedic vs. FSO	1.26 (0.91–1.74)	0.163	1.05 (0.75–1.48)	0.780
FO vs. FSO	2.57 (1.69–3.90)	<0.001	4.08 (2.44–6.82)	<0.001
Others vs. FSO	2.08 (1.22–3.53)	0.007	1.47 (0.81–2.64)	0.203
Completed first aid course	1.30 (0.90–1.87)	0.157	0.89 (0.60–1.34)	0.585

aOR—adjusted odds ratio; CI—confidence interval; FO—firefighter officer; FSO—firefighter sub-officer; ISU—Inspectorate for Emergency Situations.

## Data Availability

Data is available upon reasonable request from the corresponding author.

## Supplementary Materials

The following supporting information can be downloaded at: https://www.mdpi.com/article/10.3390/jcm15145526/s1. File S1: The Role of ISU Staff within a “First Responders” System in Romania.

## Abbreviations

The following abbreviations are used in this manuscript:

AED	automated external defibrillation
CPR	cardiopulmonary resuscitation
FO	firefighter officer
FSO	firefighter sub-officer
ISU	Inspectorate for Emergency Situations
OHCA	Out-of-hospital cardiac arrest
SMURD	Mobile Emergency Service for Resuscitation and Extrication
