# Supplementary material for: Knowledge, Attitudes, and Availability of Romanian Firefighter Paramedics to Adopt the Role of Community First Responders in Out-of-Hospital Cardiac Arrest
Source: J Clin Med. 2026 Jul 15;15(14):5526. doi: 10.3390/jcm15145526 (PMC13413123; doi:10.3390/jcm15145526)
Supplement: Supplementary file 1 [file jcm-15-05526-s001.zip › jcm-4399617-supplementary.pdf]

The Role of ISU Staff within a "First Responders" System in Romania

Research Questionnaire

Please answer a short questionnaire to assess the knowledge and availability related to providing first aid to patients in cardiorespiratory arrest within a system of "first responders". These systems, which are increasingly being developed at European level, involve the creation of groups of people who have completed basic life support courses and who agree to intervene voluntarily in their free time when they are in the vicinity of such a case, being alerted through specialized applications.

The purpose of this study is to evaluate, at a theoretical level, the possibility of voluntary involvement of ISU staff in the case of the implementation of a "first responders" type project in Romania.

The present questionnaire is addressed to the personnel (firemen/paramedics) who carry out their activity within the firefighting units of the ISU at the level of Romania. Participants have to answer a series of questions found in this form. Completing the questionnaire is the only requirement. The duration of completing the questionnaire is approximately 10 minutes.

Processing of Personal Data

In accordance with the requirements of the EU (2016/679) on the protection of individuals with regard to the processing of personal data and on the free movement of such data, the research team has the obligation to manage the personal data you provide in secure conditions. You do not need to provide your name to participate. Completing the questionnaire will represent your consent to participate in the study.

Data will be collected and stored online on Google Forms. This platform has taken the necessary actions to comply with the requirements of the EU (2016/679) on the protection of individuals with regard to the processing of personal data and on the free movement of such data.

Contact

For any questions regarding the study, you can contact the research team by email at: paul.nedelea84@gmail.com

□ By checking this box, you are stating that you agree to the above terms and that you agree to participate in the research study.

We assure you that your answers will remain confidential. The information obtained does not allow you to be identified. The statistical processing of the data provided will be analyzed at the group level and will not be presented individually in any scientific publication.

Section 1: Demographic Data

1. Age *

Answer: ____

2. Gender *

- ○ Female
- ○ Male

3. The county where I carry out my professional activity *

Answer: ____

4. Staff category *

- ○ ISU officer
- ○ ISU officer with paramedic training
- ○ ISU sub-officer
- ○ ISU sub-officer with paramedic training
- ○ Other

5. Number of years of professional experience within ISU *

Answer: ____

6. Number of years of professional experience within ISU as a paramedic *

Answer: ____

7. Have you taken and completed a first aid course? *

- ○ Yes
- ○ No

Section 2: Attitudes and Availability

8. Please indicate the degree to which you agree or disagree with the following statement: "I believe that firefighter paramedics should be involved in a system of 'first responders'." *

- ○ Strongly agree
- ○ Agree
- ○ Neither agree nor disagree
- ○ Disagree

- Strongly disagree

9. Would you like to actively participate in emergency interventions as a "first responder" in your community? *

- Yes, of course
- Yes, probably
- I'm not sure
- Not really
- Not at all

10. How prepared do you feel to deal with emergency situations as a "first responder"? *

- Very prepared
- Quite prepared
- Partly prepared
- A little prepared
- Not prepared at all

11. Would you prefer to be trained in more detail to improve your ability to act as a "first responder"? *

- Yes, of course
- I'm not sure
- Not really
- Not at all

Section 3: Open Questions

12. How do you think the integration of paramedic firefighters into a system of "first responders" could improve the time and efficiency of emergency response? (Please list a maximum of three aspects.) *

Answer: ____

Answer: ____

Answer: ____

13. What do you think would be the main obstacles or challenges to the integration of paramedic firefighters in a "first responders" system in Romania? (Please list a maximum of three aspects.) *

Answer: ____

Answer: ____

Answer: ____

Section 4: Level of Enthusiasm

14. Please rate your level of enthusiasm for the possible integration of fire-paramedics into a "first responder" system on a scale of 1 to 10, where 1 represents no enthusiasm at all and 10 represents maximum enthusiasm. *

1	2	3	4	5	6	7	8	9	10
○	○	○	○	○	○	○	○	○	○

1 = No enthusiasm at all   10 = Maximum enthusiasm

Thank you for your participation in this survey!

* Required fields
